# The Advantages of Poverty

**Published:** 1903-03-28

**Authors:** 


### THE ADVANTAGES OF POVERTY.

The disadvantages of wealth hitherto pointed out, whether as warning to the rich or as consolation of a dubiously moral sort to the possibly envious poor, have been commonly derided; since they have only been perceptible to the eye of "the trained observer." In a minor degree the same remark may apply to the disadvantage now to be discussed, but in any case it is one from which many rich people have suffered severely, though for the most part unconsciously.

When a man is sick and requires a surgical operation, poverty, paradoxical as it may seem, is to be envied, and wealth, so far at least as treatment is concerned, is a positive disadvantage. It may be well, however, before going further, to state what in this connection is intended by the term "wealth," and it will be most easily done by defining its antithesis "poverty."

The latter is any condition of life which ensures for a person a ready welcome in the wards or out-patient department ef the best hospital in his own or any other neighbourhood convenient to him; and any other condition is wealth. Now when the poor man falls sick, what does, or can, he do? He goes to his "club" or other doctor, and if what he is suffering from is an ordinary medical illness it is duly treated. If, however, it is something requiring "special" treatment or is otherwise a little bit out of the doctor's daily routine, he is promptly advised to repair to the hospital of his choice, and commonly does so. Arrived there, he either patiently awaits his turn, or, if endowed with tact and a little current coin, may secure the porter's favour and early admission to the consulting-room. But in either case he obtains that very same day the fullest attention of a consulting surgeon, and if need be is promptly admitted to a building which has been specially constructed for the reception of the sick, and wherein he will receive from surgeons and properly supervised nurses the very best treatment that the combined resources of money and science can secure for any man.

What happens to our rich man under like circumstances? He calls in the family attendant, but if anything other than medicine, etc., is required he is not always sent straight on to the specialist. The doctor naturally prefers, if he can, to treat his own patient, knows something probably about the treatment required himself, and in this case can afford time to devote to it. If successful, well and good; but if not the patient sooner or later is sent to the consultant. Arrived there he may or may not be seen the same day, but as likely as not he will be put off if the consultant is a much-sought man, and precious days of possible treatment may be lost. When, however, an operation is decided on, where is it undertaken? Practically in the vast majority of cases, unless a bed can be secured in the Home Hospital, 16 Fitzroy Square, the only choice is between the patient's own house and a nursing home; and in either, the circumstances of the rich man, both during and after operation, will compare most unfavourably with those of the poor man who requires the same treatment.

Any arrangements that can possibly be made in a private house are at the best merely makeshift, while it is doubtful, with the exception named, if there is a single nursing home in existence in which conditions are not passed which, in a hospital, surgeons would absolutely condemn. The question of expense, though in some cases an absolute catastrophe to the patient, is not here dwelt on, since the only point it is desired to emphasise is that at present the rich man with all his wealth does not, and practically cannot, obtain the scientific advantages that the poor man can, and does, obtain for nothing. Whether matters will ever right themselves remains to be seen, but there seem indications that in the not far future there will be a great increase of institutions of home or paying hospital character, and also that the essentials of the "club" system under a scheme of insurance may be so extended as practically to embrace all classes.

To many medical men the idea may be unpleasant, and while the change is going on, some no doubt will suffer. Reforms, however, which are really reforms, have an unpleasant way of effecting themselves however many toes may be trodden on. When fully developed, medical men will probably be at least as well off financially as they are now; while many would welcome in advance a condition of things which would mean more regular incomes, somewhat lessened responsibility and entire freedom from the many unpleasantnesses attaching to the system of payment per visit. In some parts of India and the Colonies a form of club payment as applied to all classes has worked happily for many years.

